# Supplementary material for: Drug repurposing for aging research using model organisms
Source: Aging Cell. 2017 Jun 16;16(5):1006–15. doi: 10.1111/acel.12626 (PMC5595691; doi:10.1111/acel.12626)
Supplement: Supplementary file 7 — Data S1 Zip‐Archive of all report cards. [file ACEL-16-1006-s007.zip › RC_ITU.pdf]

# ITU

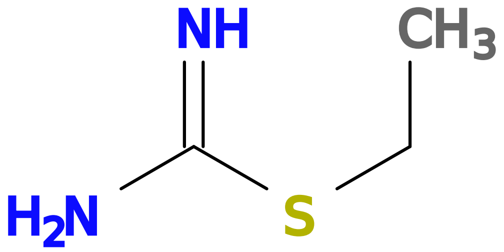


**Database identifiers**

| | |
|---|---|
| ChEMBLCompound | CHEMBL321691 |
| DrugBank | DB02234 |
| DrugBank | DB02539 |
| eMolecules | 969839 |

## Ranking

| | Rank | Score |
|---|---|---|
| Drosophila | 356/697 | 0.428 |
| C. elegans | NA | NA |

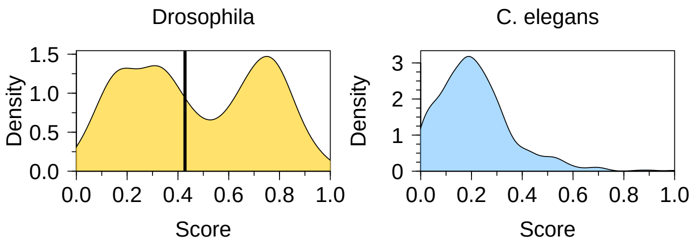


| | Ageing implication | Domain conservation | Binding site conservation | Binding affinity | Bioavailability | Lipinski | Promiscuity | Purchasability | Drug approval | **Total** |
|---|---|---|---|---|---|---|---|---|---|---|
| Drosophila | 0.624 | 0.958 | 1.0 | 0.471 | (0.9) | 0.0 | -0.0 | 0.1 | 0.075 | 0.428 |
| C. elegans | NA | NA | NA | NA | NA | NA | NA | NA | NA | NA |

## Names

- (ethylthio)formamidine
- 2-Ethyl-2-thiopseudourea
- Carbamimidothioic acid, ethyl ester
- Ethylisothiourea
- ethylsulfanylformamidine
- ethylsulfanylmethanimidamide
- Lopac-E-3149
- Lopac0$_0$00491$Pseudourea, 2 - ethyl - 2 - thio -$
- s-Ethyl isothiouronium bromide
- SEU
- Tocris-0873

## Roles

ChEBI entry None has no roles

## Status

| | |
|---|---|
| Approved drug (according to ChEMBL) | No |
| Classification (according to DrugBank) | experimental |
| Number of Rule of 5 violations | 0 |
| Binding affinity to original target in log units (RF-Score prediction) | 4.88 |
| Burns *C. elegans* bioavailability prediction | -2.12 |

## Compound Target Characteristics

### Nitric oxide synthase, inducible

Best gene implication in ageing for this target family came from gene M0RDG2 via mapping the annotation from RGD 3185 annotated in RGD 2014-03-11. Annotation GO 7568 (aging) was Inferred from Expression Pattern

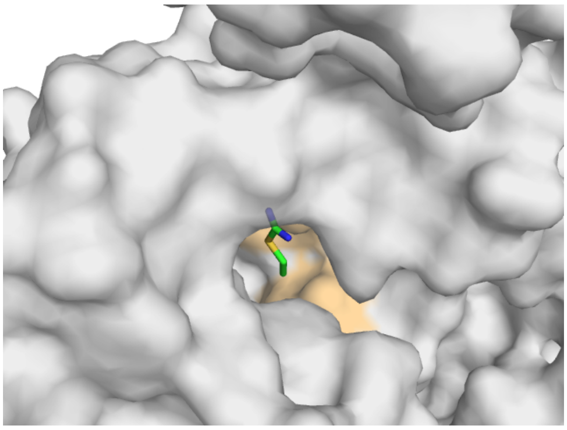

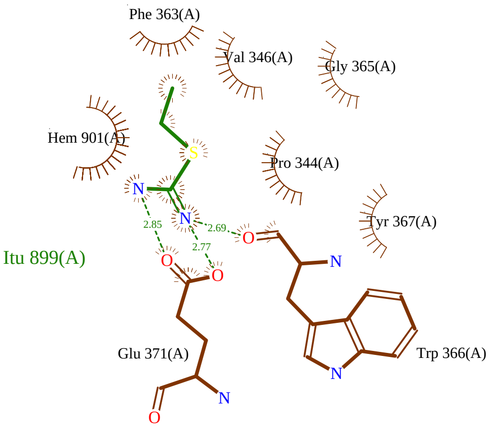


| protein | amino acids contacts (binding site) |
|---|---|
| PDB:1df1:chainA:P29477 | P V F G W Y E |
| sp:P35228:NOS2_HUMAN | P V F G W Y E |
| tr:F1LSH9:F1LSH9_RAT | P V F G W Y E |
| tr:M0RDG2:M0RDG2_RAT | P V F G W Y E |
| sp:P29477:NOS2_MOUSE | P V F G W Y E |
| sp:Q27571:NOS_DROME | P V F G W Y E |
| sp:P16603:NCPR_YEAST | - - - G D I S |

| | whole protein | | domain-based | | contact-based | |
|---|---|---|---|---|---|---|
| protein | ident | simil | ident | simil | ident | simil |
| PDB:1df1:chainA:P29477 | 1.0 | 1.0 | 1.0 | 1.0 | 1.0 | 1.0 |
| sp:P35228:NOS2_HUMAN | 0.8 | 0.93 | 0.92 | 0.98 | 1.0 | 1.0 |
| tr:F1LSH9:F1LSH9_RAT | 0.91 | 0.95 | 0.99 | 1.0 | 1.0 | 1.0 |
| tr:M0RDG2:M0RDG2_RAT | 0.92 | 0.95 | 0.99 | 1.0 | 1.0 | 1.0 |
| sp:P29477:NOS2_MOUSE | 1.0 | 1.0 | 1.0 | 1.0 | 1.0 | 1.0 |
| sp:Q27571:NOS_DROME | 0.38 | 0.68 | 0.62 | 0.91 | 1.0 | 1.0 |
| sp:P16603:NCPR_YEAST | 0.15 | 0.38 | 0.08 | 0.17 | 0.14 | 0.0 |

### Nos (FBgn0011676) associated phenotypes

chemical resistant, developmental rate defective, dominant, eclosion defective, increased cell size, large body, neuroanatomy defective, partially lethal - majority die, small body, some die during pupal stage

(Information from FlyBase)

**Nos (UniProt:Q27571) annotation**
**Function**: Produces nitric oxide (NO) which is a messenger molecule with diverse functions throughout the body. Truncated isoforms (isoform 3-isoform 6) are able to form intracellular complexes with the full length protein and serve as dominant negative inhibitors of the enzyme activity. (PubMed:11526108, PubMed:12804606, PubMed:7568075).
**Cofactor**: heme
**Cofactor**: FADNote=Binds 1 FAD. ;
**Cofactor**: FMNNote=Binds 1 FMN. ;
**Enzyme regulation**: Stimulated by calcium/calmodulin. (PubMed:7568075).
**Developmental stage**: Isoform 3 is expressed in larvae only. Isoform 4, isoform 5, isoform 6 and isoform 10 are expressed throughout development from embryos to adults. (PubMed:11526108).

(Information from UniProt)
